# Appendicitis Within an Incarcerated Inguinal Hernia Presenting as Acute Testicular Pain: A Case Report

**DOI:** 10.7759/cureus.74414

**Published:** 2024-11-25

**Authors:** Fred A Lepore, Daniel Schnick

**Affiliations:** 1 Emergency Medicine, Franciscan Health, Olympia Fields, USA

**Keywords:** acute scrotal pain, amyand’s hernia, appendicitis, case report, inguinal hernia

## Abstract

Testicular pain is a common complaint in emergency departments, with a wide range of underlying pathologies. Incarcerated inguinal hernias are an emergent and often overlooked cause of testicular pain, necessitating prompt diagnosis and management. These cases can be complex, especially when the hernia contents obscure the clinical picture. Here, we present the case of a 34-year-old male patient who presented with testicular pain and swelling, ultimately diagnosed with appendicitis within the scrotum. This appendicitis led to an incarcerated inguinal hernia requiring emergent surgical intervention. The patient had an uneventful postoperative course and remained well at the two-month follow-up.

## Introduction

Acute scrotal pain accounts for approximately 0.5% of emergency department (ED) visits and presents a diagnostic challenge due to its multiple emergent etiologies [1]. Quick recognition and treatment are vital. While common causes of acute scrotal pain include testicular torsion, strangulated inguinal hernia, and orchitis, clinicians must also consider other less frequent causes.

Appendicitis is generally thought of as an abdominal condition. Patients with appendicitis typically present with progressive abdominal pain, nausea, anorexia, and fevers [2]. Incarcerated inguinal hernias can present with both genitourinary (GU) and gastrointestinal (GI) symptoms, complicating the clinical picture. The lifetime risk of an inguinal hernia becoming incarcerated is 1-3% [3]. This case report details a patient with a chronic right-sided inguinal hernia presenting with acute scrotal pain. He was diagnosed as an incarcerated Amyand hernia caused by appendicitis, where the appendix was located in the scrotum, leading to this rare presentation.

## Case presentation

A previously healthy 34-year-old male presented to the ED with sudden-onset right scrotal pain, swelling, and erythema. Symptoms began around 8 PM and he arrived at the ED by 11 PM. He reported a history of a chronic hernia that had been asymptomatic. He denied fever, chills, abdominal pain, nausea, vomiting, dysuria, hematuria, or other symptoms.

Upon arrival, the patient’s vital signs were: temperature 36.9°C, pulse 74 beats/minute, respiratory rate 16 breaths/minute, blood pressure 160/94 mmHg, and oxygen saturation 97% on room air. Physical examination revealed a moderately enlarged scrotum with erythema, edema, and tenderness primarily over the right testicle. The abdomen was soft and non-tender, without rebound or guarding. The patient was alert, oriented, and neurologically intact.

**Figure 1:**
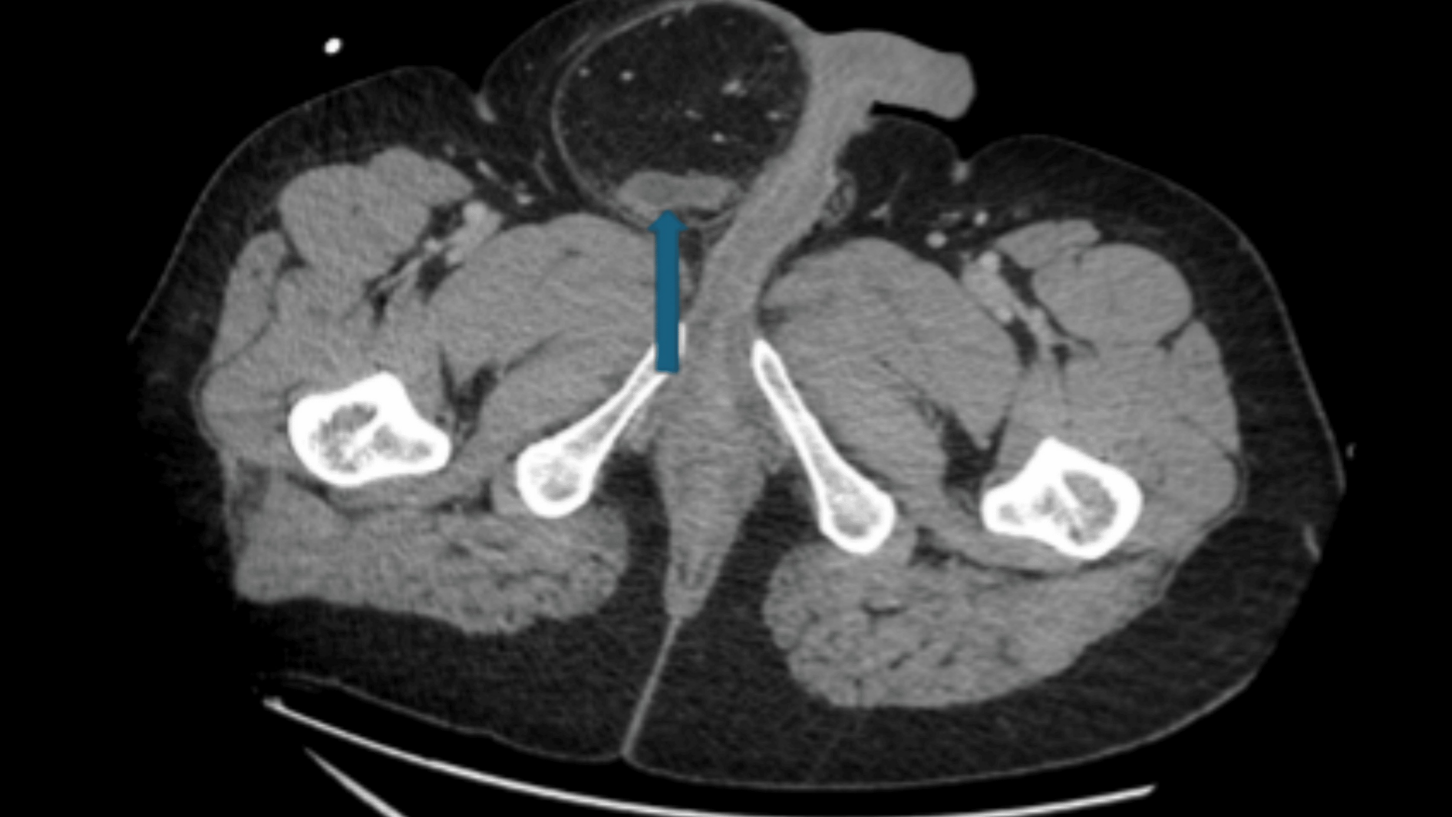
Axial contrast-enhanced CT of abdomen and pelvis demonstrating inflamed appendix within right inguinal hernia (blue arrow).

Laboratory results showed a white blood cell count of 10.60 × 10^9^/L with 85.1% neutrophils. Electrolytes were within normal limits. A contrast-enhanced CT scan of the abdomen and pelvis revealed a right inguinal hernia containing the appendix within the scrotum, consistent with an Amyand’s hernia (Figure 1). The appendix was inflamed with wall thickening and enhancement, suggestive of acute appendicitis.

The patient was started on piperacillin-tazobactam and General Surgery was consulted. The following morning, he was taken to surgery. During the procedure, the inflammation worsened and the Amyand hernia progressed to incarceration. The planned laparoscopic surgery was converted to an open procedure. The hernia sac was found to be densely adhered to the testicle. The hernia sac was carefully dissected to isolate the testicle. After the reduction of the hernia, the sac was opened to find the long, inflamed appendix adhered to the inside of the sac. The patient had an uncomplicated postoperative course. His white blood cell count normalized to 6.1 × 10^9^/L by discharge. At his two-month follow-up, the patient had no recurrence of the hernia.

## Discussion

Inguinal hernias are common in men, with many remaining asymptomatic. Despite their prevalence, the presentation of Amyand’s hernia, defined as the presence of the appendix within an inguinal hernia sac, is a rare phenomenon seen in only 0.19-1.7% of hernia cases [3]. Appendicitis within an Amyand hernia is even rarer, occurring in 0.07-0.13% of cases, often leading to delayed diagnosis [4-7].

Our patient presented with signs typical of an incarcerated inguinal hernia including scrotal pain, swelling, and erythema. While ultrasonography is often the initial imaging modality for the evaluation of scrotal pain, its sensitivity may be limited in detecting intra-abdominal pathology such as Amyand’s hernia. Cross-sectional imaging, such as CT, provides superior visualization of the hernia contents, inflammation, or complications [6]. CT imaging in this patient revealed the unexpected diagnosis of acute appendicitis within the hernia sac highlighting the importance of maintaining a broad differential diagnosis when evaluating acute scrotal pain. This condition can be deceptive as it presents without the classic signs of appendicitis such as right lower quadrant abdominal pain or nausea. Delayed treatment can significantly increase morbidity, with mortality rates for perforated appendicitis in an Amyand hernia ranging from 15-30% [5].

While Amyand hernias with appendicitis may present similarly to incarcerated inguinal hernias, prompt imaging, and surgical intervention are key to achieving positive outcomes [8]. Treatment involves antibiotics and surgery, with a reduction of the hernia and appendectomy. In our case, early administration of piperacillin-tazobactam and timely surgical intervention likely contributed to the patient’s favorable recovery.

## Conclusions

Acute scrotal pain has a broad differential diagnosis, requiring clinicians to consider both common and rare etiologies. Amyand’s hernia is a rare phenomenon, and acute appendicitis within an Amyand hernia is even rarer. This case demonstrates the importance of maintaining a high index of suspicion and promptly addressing unusual causes of scrotal pain. Prompt imaging and surgical intervention remain the cornerstone of successful management.
